# What’s Next for Modernizing Gender, Sex, and Sexual Orientation Terminology in Digital Health Systems? Viewpoint on Research and Implementation Priorities

**DOI:** 10.2196/46773

**Published:** 2023-07-25

**Authors:** Roz Queen, Karen L Courtney, Francis Lau, Kelly Davison, Aaron Devor, Marcy G Antonio

**Affiliations:** 1 School of Health Information Science University of Victoria Victoria, BC Canada; 2 Chair in Transgender Studies Unveristy of Victoria Victoria, BC Canada; 3 School of Information University of Michigan Anna Arbor, MI United States

**Keywords:** data sharing, digital health systems, digital health, gender, sex, and sexual orientation, electronic health records, GSSO, Health Information Standards, LGBT health, LGBT, policy

## Abstract

In 2021, Canada Health Infoway and the University of Victoria's Gender, Sex, and Sexual Orientation Research Team hosted a series of discussions to successfully and safely modernize gender, sex, and sexual orientation information practices within digital health systems. Five main topic areas were covered: (1) terminology standards; (2) digital health and electronic health record functions; (3) policy and practice implications; (4) primary care settings; and (5) acute and tertiary care settings. In this viewpoint paper, we provide priorities for future research and implementation projects and recommendations that emerged from these discussions.

## Introduction

Currently, most digital health systems do not have the capacity to record, store, and use accurate gender, sex, and sexual orientation (GSSO) data [1]. These outdated information practices can lead to inaccurate and potentially harmful clinical care, such as selecting the incorrect reference range for blood tests, inappropriate communication between health care providers and patients, and data invisibility for 2 spirit, lesbian, gay, bisexual, transgender, queer, intersex, asexual, and further sexual and gender identities (2SLGBTQIA+) populations [2]. Modernizing GSSO data practices and policies can have many benefits for all patients, such as improving preventive screening and affirming communication [3]. Modernization, such as updating the design of health terminology, messaging standards, and consulting with patients, is underway in many jurisdictions [4,5]. However, there is much more work that needs to be done to create digital health systems that fully, accurately, and safely incorporate modernized GSSO data to better serve the needs of health care stakeholders [1].

In 2021, people who were affected by and interested in participating in modernizing GSSO data practices engaged in a series of web-based discussions with the goal of sharing the Canadian Action Plan for modernizing GSSO data in electronic health records (EHRs) [1,5]. The action plan adapted the Equity-Oriented Health Care Intervention (EQUIP) framework as the theoretical foundation for informing how to address the current fragmentation and inaccurate GSSO data by enhancing organizational (including technical) capacity for equity-oriented design [6,7]. Through our adaptation of the EQUIP intervention, we illustrated how modernizing GSSO terminology and EHR systems can contribute to trauma-and-violence-informed care, harm reduction, and culturally safe care. One of the goals of these discussions was to create a roadmap for context-specific implementation planning and further research. Invitations were sent to community groups and organizations involved with 2SLGBTQIA+ advocacy and service provision to invite 2SLGBTQIA+ people, clinicians, informaticists, terminology specialists, policy makers, and vendors to participate in a series of web-based engagement sessions [5]. This protocol was reviewed and approved by the University of Victoria’s Human Ethics Research Board (21-0109). The number of participants at these sessions ranged from 11 to 18 people. Each session focused on 1 of 5 main areas: terminology [8,9]; digital health and EHR functions [10,11]; policy and practice implications [12,13]; primary care settings [14]; and acute and tertiary care settings [15]. This viewpoint paper provides priorities for future research and implementation projects and recommendations that emerged from these sessions.

## Terminology Priorities and Progress

Although the sessions mentioned above took place in Canada, the digital health information systems that are used rely on international terminology standards, such as Systematized Nomenclature of MEDicine-Clinical Terms (SNOMED CT), Health Level 7 International (HL7), and Logical Observation Identifiers Names and Codes (LOINC). SNOMED CT is the most comprehensive controlled health terminology in the world and is an international interoperability standard [16,17]. Changes to the design of technical artifacts, such as clinical data elements and messaging standards, affect how GSSO data are represented, exchanged, and stored and have broad implications for health organizations. Some areas affected will include health insurance, medical billing systems, legal systems, and health care cultures [2]. For example, many health care organizations use a government ID for identity verification when a patient receives services. With current, outdated systems, a patient’s clinical information (sex or gender) may not match the government ID, such as a driver’s license or passport. This mismatch may lead to clinical information being overwritten by administrative data from a non–health organization in order to resolve the discrepancy and facilitate billing based on the ID card. But overwritten clinical information may create new mismatches between provided clinical services and billing business rules, such as those limiting reimbursement for cervical cancer screening to patients with a recorded sex of female. This would mean that the charge for cervical cancer screening for a transgender man with a cervix may be rejected by insurance systems. Modernizing the design of health information systems will help eliminate these types of issues. The initial terminology sessions prompted further work on both the Canadian national and international editions of SNOMED CT [4].

Some participants in our sessions and members of the research team were concurrently involved with the work in the HL7 Gender Harmony Project (GHP) [17]. HL7 standards are messaging standards and are in wide use internationally. Updates to HL7 messaging standards affect data interoperability, which is the exchange of standardized data between digital health systems. In Fall 2022, proposed changes to HL7 messaging standards arising from the GHP were balloted, and work continues to resolve community comments. These proposed changes are significant because these standards will affect nearly every digital health system in Canada [16]. Fast Healthcare Interoperability Resources (FHIR), a backbone in mobile health application development, is one of several HL7 standards being modernized. Therefore, the HL7 updates will affect consumer health systems as well as clinical health information systems.

Further community consultation on anatomic and hormone inventories was identified as a priority for future research and implementation planning [8,9,12,13]. In discussions about GSSO information practices in primary care and acute care settings, the use of inventories and appropriate data collection were raised [14,15]. In 2021, the concept of “Sex for Clinical Use” (SFCU) was being developed by the HL7 GHP to separate administrative sex elements from clinical ones [17]. SFCU could link to patient-centric anatomic or hormone inventories. The intention was that SFCU would be a method for clinicians to indicate special considerations for testing, diagnoses, or treatments irrespective of the datum recorded in a sex or gender identity field. For example, a clinician could use this to indicate that for laboratory blood tests, a female reference range should be used regardless of the recorded sex or gender of the patient.

In discussions following this project, the Canada Health Infoway Sex and Gender Working Group (SGWG) has recommended the implementation of patient-centric anatomic and hormone inventories. This inventory approach is currently being discussed by HL7 as part of the GHP balloting process [17]. Additional use cases, case studies, and examples are needed for future research and implementation. Building these examples will require the expertise of clinicians, 2SLGBTQIA+ patients with lived experience, as well as informaticists and terminology specialists.

Support for GSSO data in additional languages other than English and perhaps extensions of terminology standards for indigenous populations were also identified as areas for future exploration.

## Understanding Digital Health and EHR Function Priorities

Participants noted identity verification and data prioritization as key areas for further exploration [10,11]. Commonly, recorded sex or gender is used within the registration process as a second verification method for identity beyond a health services card. This can lead to conflicts between current gender identity information and information from government-issued forms of identification that may not be up to date or be able to be updated [10,12]. Although participants recognized how identity theft and resulting fraud are concerns for patients and providers, participants suggested that other means for identity verification should be explored but alternative means were not discussed in depth.

Participants also wanted prioritization of data to be addressed [10,11]. In some existing digital health systems, administrative data, such as recorded sex or gender, may be prioritized and may overwrite updated clinical information. This can lead to inappropriate, possibly harmful, clinical care based on erroneous assumptions about the presence or absence of organs or the use of inappropriate laboratory reference ranges. Likewise, overwriting updated clinical information may lead to misgendering, which is detrimental to respectful clinician-patient relationships and trust [13,15].

More work with digital health information system vendors to update existing systems was recommended by participants [10,11]. Changes to international standards such as SNOMED CT and HL7 are likely to provide incentives for vendor updates [1,2]. Since the initial sessions, the SGWG has been consulted several times by a Canadian EHR vendor as they update how their EHR collects, organizes, and shares GSSO data [18].

For implementation planning, participants suggested that organizations should perform environmental scans to understand what information is being collected within their information systems and how it is being used and reused within these systems [10,11]. This exercise would be particularly helpful in understanding how GSSO changes may propagate through digital health information systems and could allow analysts to proactively address exchange issues before implementation. Participants suggested that sharing these scans could have many benefits [11].

Participants also noted that GSSO information modernization cannot be considered in isolation from other types of sociodemographic or clinical information held within digital health systems. Intersectionality is an analytic lens that is used to investigate structural forms of marginalization, such as race, gender, and sexual orientation [19,20]. A focus on a single factor may lead to not fully understanding all of the structural barriers or how factors may interact with one another. For example, the level of stigma or discrimination a transgender woman may experience may be complicated by other factors such as race or class. More exploration of intersectionality, particularly with people with 2SLGBTQIA+ lived experience, is needed [2,12,13].

Discussions about reuse of data and intersectionality prompted a recommendation from participants for more consultation with affected parties regarding data aggregation versus visibility [10-14]. Historically, data from populations with small numbers have been aggregated into larger categories by analysts in order to avoid possible individual identification. Aggregated categories have often not been created in consultation with affected communities [2]. There have been calls for greater representation of 2SLGBTQIA+ experiences in health outcome reporting [3]. While reporting at a national level may not have implications for individual reidentification, this may be an issue at a provincial, territorial, or health authority level. People who are most affected by data aggregation and underrepresentation need to be partners in finding an appropriate balance between visibility and aggregation [2].

## Context-Specific Priorities

During our discussions, participants noted that the variety of practices that exist in health care are dependent upon unique health care contexts. Below, we detail specific considerations for these different health care settings.

For primary care settings, participants noted the variety of physical space and environmental design as concerns for GSSO data collection [14,15]. For example, the physical layout of desks within the patient registration area may be a barrier to patient confidentiality and privacy. While other clinics’ practices such as calling out a patient name with an honorific (eg, Mr., Ms., or Mx.) or displaying names on message boards may be inappropriate. Participants suggested exploring the use of self-reporting mechanisms for data collection, such as kiosks or smart devices [10,11]. In addition, it was noted that policies also need to be developed for appropriate information display in postencounter communications, (eg, envelopes or phone messages) [15].

For acute care settings, participants noted that the timing and place of GSSO data collection were an important implementation consideration [14]. For example, during an emergency department visit, participants suggested that pronouns may be collected during registration, but more sensitive GSSO information such as gender identity and anatomic inventory information should be collected during triage with a health care provider [14]. This information may affect services such as bed assignment or radiology exams (eg, x-rays or ultrasounds), which may be performed at the bedside, and therefore the information may need to be collected before seeing a physician. Unique to the acute care setting, participants also recommended further examination of minimum data-sharing standards for unconscious patients.

The use of remote health care visits requires a greater understanding of best practices for digital care and GSSO data collection and use. In a remote visit, the physical space expands to include the patient’s space, and GSSO policies and practices need to recognize this inclusion [10,11]. While digital care expands the potential for web-based data collection, participants identified language, digital health literacy, and technology access as potential barriers [8-11,14,21].

In both primary and acute care settings, participants noted that referrals may be made for additional screening, diagnoses, or treatments [14,15]. This led to concerns about patient control of information shared between different health care providers and the potential for masking nonrelevant GSSO data in the referral process [13,14]. In addition, participants noted that not all health services are provided within a health care facility. Some services, particularly mental health and harm reduction services, are provided by community service agencies [14,15]. This needs to be considered when developing policies regarding sharing of data between agencies [10,11].

## Developing Use Cases and Policies for the Social and Health Care Context

Participants recommended further development of use cases for intake, registration, clinical encounters, and referrals in order to understand how and when GSSO data should be collected and used [10-13]. Data mapping illustrates how data are used and reused within an information system and when exchanged between systems [10,11]. As part of use case development, participants suggested further work in mapping specific data fields to work processes in order to facilitate organizational policy development and health outcomes reporting [10-13]. Participants supported the idea that guidelines for minimum data collection standards for different types of encounters should be developed in collaboration with patients and clinicians [14,15]. Participants noted that because of the high degree of internal data sharing between different services within an acute care facility, a better mapping of GSSO data and acute care workflow for internal data sharing is needed.

Patient portals were provided as a possible example of how patients can control access to specific information when they choose to share portal access with others. However, it was noted that policies regarding the masking of data may be particularly important for youth receiving care, as they may wish to control proxy or parental access to certain types of information within their patient portal [14,15].

## Cross Cutting Recommendations for Culturally Competent GSSO Data Collection

**Figure 1:**
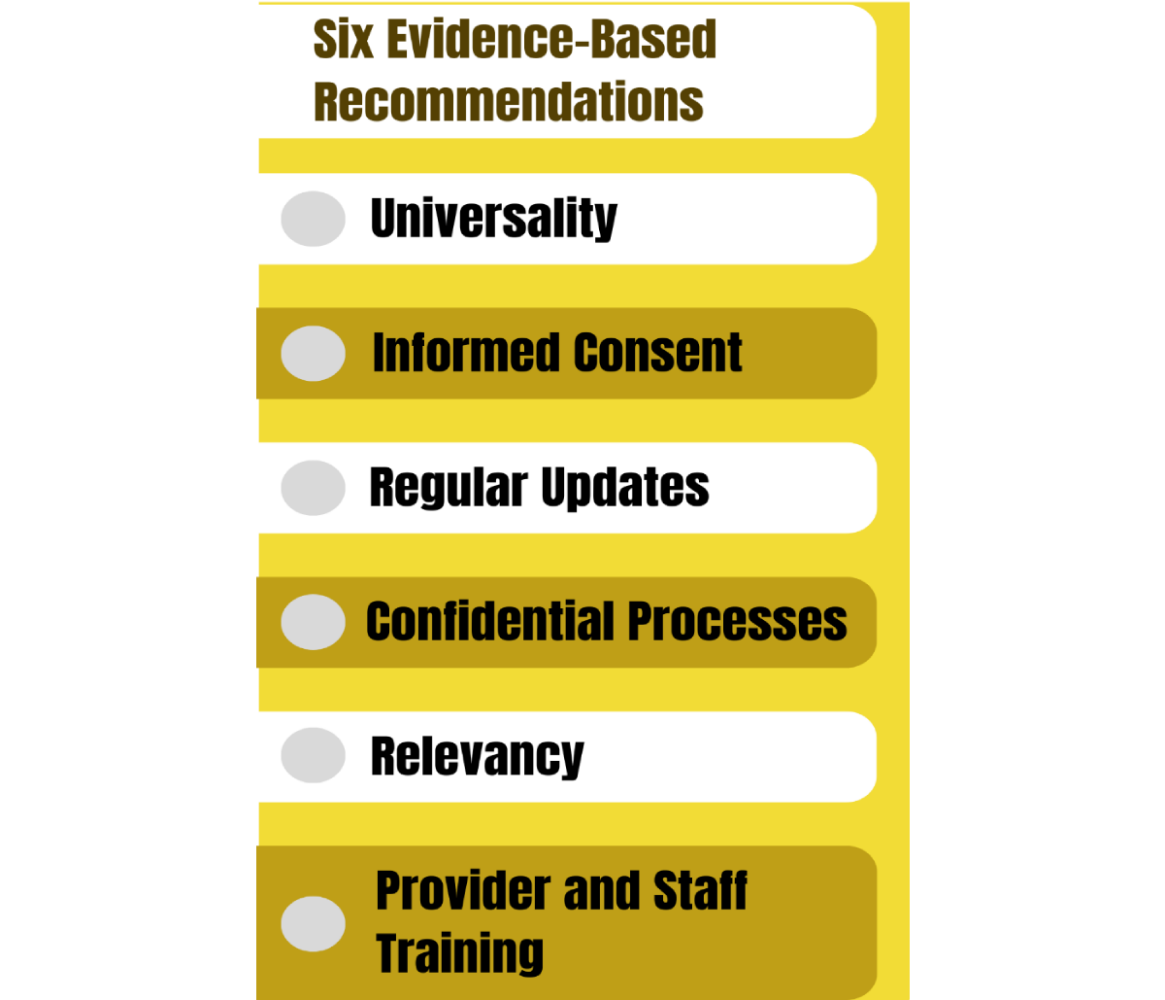
Six evidence-based recommendations for gender, sex, and sexual orientation data collection.

Across the discussions, six evidence-based recommendations were reaffirmed for collecting GSSO data: (1) universality; (2) informed consent; (3) regular updates; (4) confidential processes; (5) relevancy; and (6) provider and staff training (Figure 1) [2,22]. Data collection should be universal, meaning that GSSO data should be collected from all patients. Informed consent means that patients need to know why questions are being asked and how their data will be used [22]. GSSO information should be routinely updated at least once a year or when errors are noted. The process of data collection should be confidential using standardized forms that ideally are self-administered, such as through a patient portal [23]. If using paper-based forms, additional consideration will need to be given to who will enter the data [12,15]. The verbal collection of data needs to be done in a private setting. Any GSSO information requested should be relevant to the patient’s clinical encounter [24,25]. All staff and clinicians should be trained on how to ask for GSSO information in a culturally safe manner [22,26].

## Next Steps and Conclusion

Modernizing GSSO information practices involves many technical, clinical, work processes, societal, and ethical challenges (see Table 1). The complexity of health care technologies and processes may be barriers that deny access to care [8]. Additional design considerations are needed to ensure that health information systems enable access to quality care for patients and support clinicians with the information they need to provide quality care.

Technical challenges include continuous updates to international standards and ensuring that interoperability between digital health information systems within and among organizations is promoted [10,11]. While this work may be done primarily by standards specialists and health informaticists, clinicians and health policy leaders must also be involved, particularly in regard to interoperability and data sharing issues.

Some of the clinical challenges that will likely arise with GSSO modernization could include creating and maintaining affirming environments and training and support for clinical staff to have respectful, safe, and meaningful clinical encounters with patients [12-15]. Clinicians will likely need to be engaged in informing clinical encounter and referral use cases and in furthering our understanding of how and when GSSO data should be collected and used [13-15]. This feedback could likely be valuable to ensure accurate clinical information is readily available for decision-making [14,15].

Modernizing GSSO information practices will likely also affect work processes within health care environments along the continuum of care. Workflows should be designed with modern GSSO policies and practices for collecting or sharing information in mind [12,13]. These workflow changes will not just be clinical in nature because the supportive work done by other staff is likely to be affected by these changes as well. Administrative and clerical staff could also require training, education, and support for new work processes and policies [12,13]. For example, bed or room assignment processes may be affected [15]. Physical environments may need alterations to protect privacy during GSSO data collection.

This work may also pose broader challenges for health care in that it may necessitate a rethinking of the role of patients in regard to controlling their own information and the manner in which different populations are represented in health outcomes reporting [12,13]. In addition, strategies will likely need to be developed to ensure the quality of data. Patients might not be comfortable reporting information about GSSO with clinicians until trust is established [2,22]. Therefore, education about how this data will be used will likely be required at both the patient- and clinician-level [26]. Benefits will most likely be far-reaching. The use of anatomic or hormone inventories could result in more accurate screening, treatment, and recommendations for all patients [25]. For example, using the recorded presence of a cervix in an anatomic inventory can more accurately target cervical cancer screening resources. Likewise, anatomic inventory data regarding the presence or absence of a prostate is important in providing the correct laboratory reference ranges for prostate-specific antigen results. When information regarding a prostatectomy is missing, incorrect prostate-specific antigen reference ranges may be used, resulting in a missed diagnosis of metastatic prostate cancer.

As with any systemic change, one must be vigilant for unintended consequences, and modernizing GSSO information and practices is no different. Our work has limitations in regard to who were invited to and who had the time and energy to participate in our meetings given individual constraints. Although our participant sample may not be fully representative of impacted stakeholders, we included all who expressed interest. While there may be an assumption that GSSO modernization will be beneficial for only some patient populations, the reality is that these types of changes will likely have benefits for all patients as they will receive more individualized care [2,21].

**Table 1:** Summary of identified barriers to modernizing gender, sex, and sexual orientation (GSSO) data practices as well as suggested research and interventions to address these barriers.

Topic area	Identified barrier to modernizing GSSO data practices	Suggested research or intervention to address barrier
Terminology	Outdated health information standards	Update standards Ballot new terminology Community consultation
Terminology	Lack of rich data to serve stakeholders’ needs	Patient-centric anatomic and hormonal inventories Development of use cases, case studies, and examples
Digital health and EHR^a^ functions	Patient identification and verification	Explore other means for identity verification
Digital health and EHR functions	Data prioritization and overwriting of updated data	Work with EHR vendors and Health Information Standards Organizations to update existing systems Work with jurisdictions for compatible standards for interoperable non–health information systems
Digital health and EHR functions	Implementation challenges	Environmental scans Community consultations
Context-specific priorities: primary care	Privacy	Self-reporting mechanism for data collection Redesign of physical spaces Policy development
Context-specific priorities: acute care	Timing and location of data collection	Policy development and clinical guidelines Review of minimum data sharing standards
Context-specific priorities: remote care	Lack of understanding of best practices	Development of GSSO policies and practices
Context-specific priorities: referrals	Patient control of information	Mask of nonrelevant GSSO data Community consultation
Clinical scenarios	Lack of understanding of how and when GSSO data should be collected and used	Use case development Data mapping exercise Guideline development for minimum data collection standards Setting-specific workflow analysis

^a^EHR: electronic health record.

## Abbreviations

2SLGBTQIA+: 2 spirit, lesbian, gay, bisexual, trans, queer, intersex, asexual, and further sexual and gender identities
EHR: electronic health record
EQUIP: equity-oriented health care intervention
FHIR: Fast Healthcare Interoperability Resources
GHP: Gender Harmony Project
GSSO: gender, sex, and sexual orientation
HL7: Health Level 7
LOINC: Logical Observation Identifiers Names and Codes
SFCU: sex for clinical use
SGWG: Canada Health Infoway Sex and Gender Working Group
SNOMED CT: Systematized Nomenclature of MEDicine-Clinical Terms
